# Multi-decadal ocean temperature time-series and climatologies from Australia’s long-term National Reference Stations

**DOI:** 10.1038/s41597-022-01224-6

**Published:** 2022-04-07

**Authors:** Moninya Roughan, Michael Hemming, Amandine Schaeffer, Tim Austin, Helen Beggs, Miaoju Chen, Ming Feng, Guillaume Galibert, Clive Holden, David Hughes, Tim Ingleton, Stuart Milburn, Ken Ridgway

**Affiliations:** 1 Coastal and Regional Oceanography Lab, Centre for Marine Science and Innovation, UNSW Sydney, Sydney, NSW 2052 Australia; 2 School of Mathematics and Statistics, UNSW Sydney, Sydney, NSW 2052 Australia; 3 Science & Innovation Group, Bureau of Meteorology, Docklands, Vic 3008 Australia; 4 CSIRO Oceans and Atmosphere, Indian Ocean Marine Research Centre, Crawley, WA 6009 Australia; 5 Australian Ocean Data Network, Integrated Marine Observing System, UTAS, Hobart, TAS 7001 Australia; 6 Oceanographic Field Services, Freshwater, NSW 2096 Australia; 7 CSIRO Marine Laboratory, Battery Point, TAS 7004 Australia; 8 NSW Department of Planning Industry and Environment, Lidcombe, NSW 2141 Australia

**Keywords:** Climate change, Physical oceanography

## Abstract

Multi-decadal ocean time-series are fundamental baselines for assessing the impacts of environmental change, however, compiling and quality controlling historic data from multiple sources remains challenging. Here we aggregate, document, and release a number of long time-series temperature products and climatologies compiled from data obtained at 4 monitoring sites around Australia where sub-surface ocean temperature has been recorded nominally weekly to monthly since the 1940s/50s. In recent years, the sampling was augmented with data obtained from moored sensors, vertical profiles and satellite-derived data. The temperature data have been quality controlled, and combined using a rigorously tested methodology. We have packaged the multi-decadal, multi-depth, multi-platform temperature time-series at each site and produced a range of daily temperature climatologies from different data combinations and time periods. The 17 data products are provided as CF-compliant NetCDF files and will be updated periodically. The long-term temperature time-series will be useful for studies of ocean temperature variability, trends, anomalies and change. The data collection is supported by Australia’s Integrated Marine Observing System and data are open-access.

## Abstract (summary)

**Table:** 

Measurement(s)	temperature of water • temperature of sea surface
Technology Type(s)	ocean time series station • satellite • ship-based water sampling • ship-based CTD profiles
Sample Characteristic - Environment	coastal sea water • sea water environment • continental shelf
Sample Characteristic - Location	Australia • Tasman Sea coastal waters of continental Australia • Western Australia

## Background & Summary

Long-term ocean temperature time-series have become increasingly important as baseline data^1,2^ that can reveal the extent of the ocean response to climate change, particularly ocean warming^3–6^. Long time-series are needed to create climatologies (requiring multi-decadal data) that can provide insight into shorter timescale marine extremes and anomalies such as heatwaves^7,8^ and cold spells^9^ and to show ocean warming trends, which are non-uniform globally^2,10^.

The satellite era has provided us with near global surface ocean temperature data with which to derive baselines and to assess change at the sea surface^11,12^. Notable sub-surface ocean time-series include the Hawaii Ocean Time-series (HOTS^13^) and the CalCOFI surveys^14^. However sub-surface, particularly full water column ocean time-series are rare, yet are becoming increasingly important, for example, recent studies have shown that extreme warming events (e.g marine heatwaves) are underestimated by satellite data in both peak and intensity^8,15^. Multi-decadal data-sets provide a valuable global benchmark for tracking ocean health, particularly for ecosystems at depth and an experimental framework for studying seasonal and inter-annual ecosystem dynamics.

**Fig. 1:**
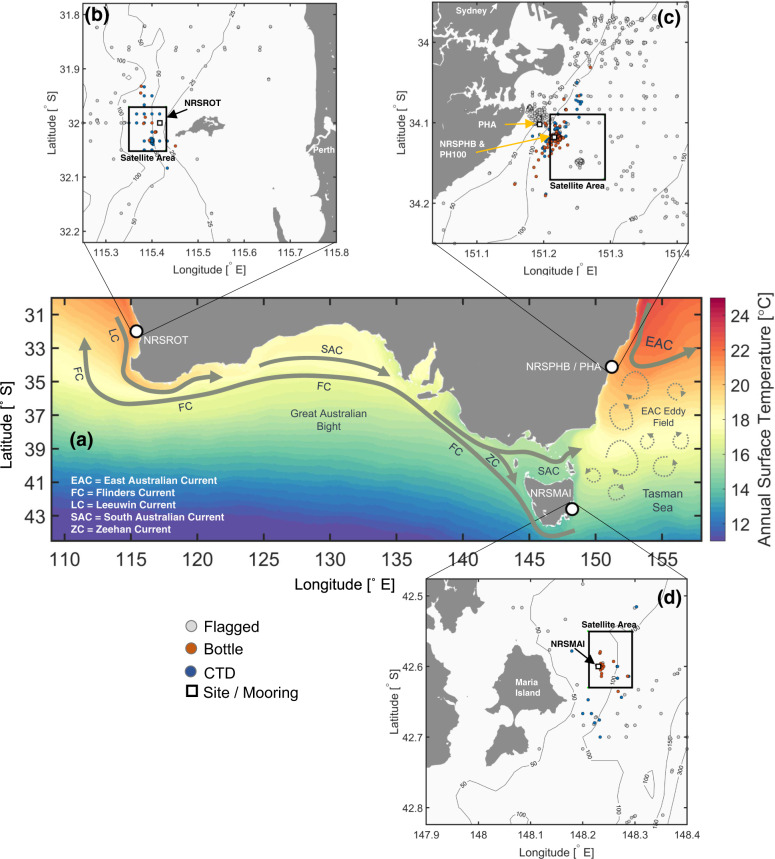
Map and schematic diagram showing the locations of the data collection around Australia. (**a**) Annual mean sea surface temperature provided by the SSTAARS climatology data product^11^ with typical ocean circulation patterns superimposed as grey arrows. The locations of the data collection sites: (**b**) Rottnest Island (NRSROT), (**c**) Port Hacking (PHA and NRSPHB/PH100), and (**d**) Maria Island (NRSMAI). The positions of the data: bottle (orange spots), CTD casts (blue spots) and moorings (white square) data are shown, alongside nearby data flagged as not usable (grey spots). The NRSPHB/PH100 and PHA sites are included in panel (**b**), but only the bottle and CTD data used for the climatology at NRSPHB/PH100 is coloured. The black boxes in panels (**b**-**d**) indicate the area over which satellite SST data were averaged. Map information sourced from Google: Map data ©2021 Google.

Australia has some of the longest sub-surface oceanographic time-series in the world. Nominally weekly biogeochemical sampling commenced in the 1940s at four coastal locations around Australia (Fig. 1) and has been continued at weekly to monthly intervals since. Sampling was boat-based initially and used bottles to collect water at discrete depths. In recent decades the bottle sampling was augmented with approximately monthly vertical profiles from an electronic CTD (conductivity, temperature, depth) sensor, and at some sites a moored thermistor array and/or CTD sampling at high temporal resolution at multiple depths. Concurrently, satellite-derived surface data has increased in availability and resolution. These data provide a unique insight into the mean, variability, extremes and trends in coastal ocean properties over the past 7–8 decades.

Historic ocean data (spanning multiple decades) can be challenging to find and access. It is not uncommon for multiple versions of long-term data-sets to be stored on local machines, floppy disks or hexabyte tapes, and to be lacking version control. Quality control (QC) may have been done on some aspects of the data, and other QC may have to be redone every time the data is shared. The Integrated Marine Observing System (IMOS, www.imos.org.au) has led provision of open-access data in Australia through the Australian Ocean Data Network and their data portal, which provides access to standardised data-sets stored centrally. Here, we improve on this through the provision of rigorously QC’d version controlled compiled long-term temperature data-sets. By adherence to FAIR data principles^16^ it is our intention that we will minimise the need for other stakeholders to go through the arduous process of collating, QC’ing and aggregating these valuable data.

Additionally, after the data have been collated, QC’d and aggregated, combining multiple data-sets into data products increases their utility, but can also be a challenge. These data-sets can include different data collection methods (water samples, electronic vertical profiles and *in situ* records), a range of sampling depths and locations, different sampling times (time of day, month, year) and sampling frequency (ranging 5 minute to monthly or longer). However, it is very important to combine the data-sets in way that avoids aliasing or biasing^17^.

This study was initially motivated by the need for a robust sub-surface ocean climatology with which to identify marine extremes such as marine heatwaves and cold spells, (defined as extreme deviations from a 30-year climatology^7^). Through the development of open access robust data products including climatologies and percentiles we will alleviate the need for others to repeat our efforts and ensure robustness. These data will be useful for anyone wanting ready access to temperature time-series from Australia’s long-term national reference stations (NRS), and derived data products that include daily temperature climatologies (including mean, median, standard deviation and percentiles) at a range of depths. The paper also serves as an example methodology for other data stewards.

We present 17 data products from four sites around Australia (Fig. 1): Port Hacking A (PH050), Port Hacking B (PH100), Maria Island on the east coast and Rottnest Island on the west coast. The numerous data products are provided as Climate Forecasting (CF) compliant NetCDF files with an accompanying digital object identifier (DOI), and example code is given for accessing and plotting the data in MATLAB, Python, and R. The data are obtainable from the Australian Ocean Data Network (AODN) thredds catalog.

## Methods

### The study sites

Sub-surface ocean data have been collected at 4 sites around Australia since the 1940/50 s. There are two sites off Port Hacking, Sydney (~34°S) in approximately 50 m and 100 m of water (Fig. 1c). These were originally named PHA and PHB and were started in 1942 and 1953 respectively. PHB was incorporated into the IMOS National Reference Station (NRS) network in 2009^18^ (see below), and was renamed NRSPHB. There is a mooring deployed near the site of NRSPHB, named PH100^19–21^.

Off the southeast coast of Tasmania is the Maria Island (MAI) NRS (NRSMAI, ~42.8°S) in approximately 90 m of water (Fig. 1d), which has previously been referred to as the ‘Maria Island time-series’ (MITS)0^6,22^. Off the west coast, the NRS site is west of Rottnest Island (NRSROT ~ 32°S) off Perth, Western Australia, in approximately 55 m of water (Fig. 1b).

These sites are the longest sub-surface ocean time-series in the southern hemisphere and are the backbone of Australia’s NRS network; a network of long-term sampling sites (currently 7) around Australia. The time-series were started by Australia’s Commonwealth Scientific and Industrial Research Organisation (CSIRO), and championed by ex-chief of CSIRO Fisheries and Oceanography, David Rochford^23,24^. Sampling commenced in 1942 at PHA, and gradually expanded regionally, supported by local government agencies. Since 2009 the data have been maintained through IMOS as a core component of Australia’s national ocean observing effort^18^. These data have been used in a number process studies over the years and for investigating long-term environmental change^1,4,6,22,25^.

**Fig. 2:**
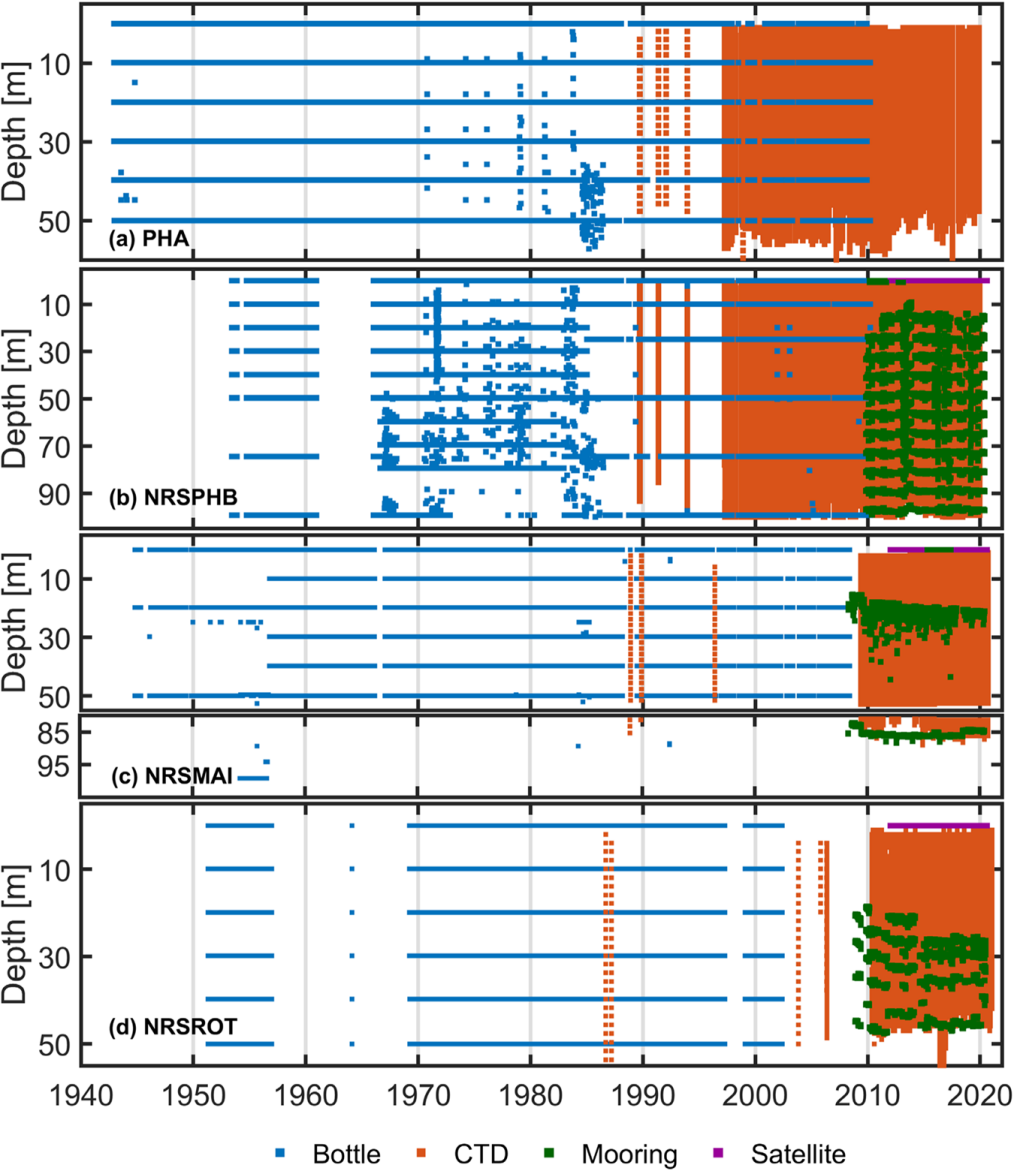
Time-series showing data availability by platform (bottle, CTD, mooring and satellite data) by depth at the four stations: (**a**) PHA, (**b**) NRSPHB, (**c**) NRSMAI, and (**d**) NRSROT. Note the depth ranges change for each site, and for (**c**) depth range has been split into two: 0–55 m and 80–90 m depth showing the availability of near bottom data, primarily commencing with the mooring deployment in 2009.

Compiling metadata from historic data-sets is challenging, thus we draw on a number of studies notable for their robust metadata^1,4,18–21,26,27^. The compiled metadata are shown in Table 1 including location, depth, sampling characteristics, and time range. The data availability at each site is shown in Fig. 2 as a function of time and depth.

**Table 1:** The metadata after QC for all *in situ* data platforms accessed for the data products. ﻿Includes site locations, sampling depths, distance offshore, sampling platform (Bottle, CTD or Mooring), sample depths, frequency, and the record start and end dates at each site: Port Hacking (PHA, PH050 and NRSPHB/PH100), Maria Island (NRSMAI) and Rottnest Island (NRSROT).

Site Location, State and name		Latitude range (° S)	Longitude range (° E)	Max. Sample Depth (m)	Approx. distance Offshore	Sampling Method	Typical Nominal Depths (m)	Typical frequency	Start Date	End Date
Port Hacking 50 m, New South Wales PH050	PHA	34.15 to 34.02	151.15 to 151.25	57	5.5 km (east of coast)	Bottle	0, 10, 20, 30, 40, 50	weekly - fortnightly	Dec 1942	Mar 2010
		34.16 to 34.02	151.12 to 151.25	60		CTD Casts	Full depth (1 m intervals)	weekly - monthly	Apr 1997	Dec 2019
Port Hacking 100 m New South Wales NRSPHB/PH100	PHB PH100	34.08 to 34.07	151.25	100	8.5 km (east of coast)	Bottle	0, 10, 20, 30, 40, 50, 75, 100	weekly - fortnightly	May 1953	Dec 1960
		34.11 to 34.05	151.18 to 151.25	100		Bottle	0,10, 20, 30, 40, 50, 60, 70, 80, 100	weekly	Jan 1966	Jan 1985
		34.19 to 34.07	151.19 to 151.26	100		Bottle	0, 10, 25, 50, 75, 100	fortnightly- monthly	Jan 1985	March 2010
		34.19 to 34.03	151.16 to 151.27	100		CTD Casts	Full depth (1 m intervals)	fortnightly- monthly	Apr 1997	Dec 2019
		34.12	151.22	115	8.5 km (east of coast)	Mooring (Sub-surface)	8 m intervals (15–111 m)	5 minute	Oct 2009	May 2020
		34.12	151.22	0.6		Mooring (Surface)	0.6 m	5 minute	Feb 2010	Sep 2011
		34.12	151.22	0.6		Mooring (Surface)	0.6 m	5 minute	Sep 2012	Mar 2013
		34.13 to 34.1	151.21 to 151.23	100		CTD Casts	Full depth (1 m intervals)	monthly	Feb 2009	Dec 2019
Maria Island 90 m, Tasmania NRSMAI	MAI	42.6	148.27	105	7.5 km (east of Maria Island), 24 km (east of coast)	Bottle	0, 20, 50	fortnightly - monthly	Oct 1944	Oct 1956
		42.70 to 42.52	148.18 to 148.30	89		Bottle	0, 10, 20, 30, 40, 50	fortnightly - monthly	Oct 1956	May 2008
		42.64 to 42.58	148.23 to 148.29	89		CTD Casts	Full depth (1 m intervals)	monthly	May 2009	Sep 2020
		42.6	148.22	89		Mooring (Sub-surface)	20 m, 88 m	15 minute	Apr 2008	May 2020
		42.6	148.22	0.6		Mooring (Surface)	0.6 m	hourly	Apr 2015	May 2017
Rottnest Island 55 m, Western Australia NRSROT	ROT	32	115.42	50	6 km (west of Rottnest Island), 33 km (west of coast)	Bottle	0, 10, 20, 30, 40, 50	fortnightly	Apr 1951	Dec 1956
		32.08 to 31.93	115.35 to 115.43	57		Bottle	0, 10, 20, 30, 40, 50	weekly - monthly	Mar 1969	May 2002
		32.04 to 31.93	115.38 to 115.45	58		CTD Casts	Full depth (1 m intervals)	fortnightly - monthly	May 2010	Jan 2021
		32	115.4	50		Mooring (Sub-surface)	22, 27, 33, 43	5–15 minute	Nov 2008	Mar 2014
		32	115.4	45		Mooring (Sub-surface)	43–45	5–15 minute	Mar 2014	Aug 2014
		32	115.4	62		Mooring (Sub-surface)	24, 33, 43, 55	5–15 minute	Aug 2014	Jul 2020

### Boat-based temperature sampling (Bottle and CTD)

*In situ* water samples have been collected since the beginning of the records (1940/50 s), originally with Nansen and then Niskin bottles. Properties collected typically included temperature, salinity, dissolved oxygen concentrations, nitrate, phosphate, and silicate^1^ at fixed depths/pressures. Here we only use temperature (T) and pressure (P) records, however, the methodology may be suitable to expand to other variables, such as salinity, in the future.

Initially, T was measured with reversing thermometers and readings were corrected for thermal expansion with an estimated accuracy of better than ±0.02 °C^1^. Accurate positional information was challenging if not impossible to obtain prior to GPS technology, hence, early sample locations are approached with caution and additional QC procedures are followed (see section on QC below). The sampling time of day is also unreliable prior to 1996 and only a date (not time) is available for a large portion of the earlier records, although it is known that samples were generally taken in the morning.

Regular *in situ* boat-based sampling typically varied between weekly to monthly (Tables 1, 2) with the number of yearly samples varying between less than 10 and over 50. Currently boat-based sampling is nominally monthly, with 11–12 samples per year at each site. The samples have typically been processed at the CSIRO Marine Laboratory (from 1944–1984, in Cronulla, New South Wales and from 1984 in Hobart, Tasmania) with support from state agencies, such as the New South Wales Department of Planning, Industry and Environment (formerly the Office of Environment and Heritage).

**Table 2:** A summary of the temporal data coverage for each of the data sets by platform (Bottle, CTD, Mooring, Satellite) at each site: Port Hacking (PHA/PH050 and NRSPHB/PH100), Maria Island (NRSMAI), and Rottnest Island (NRSROT). Also shown are the coverage percentages for the Aggregated and Gridded data products. ‘Year days with data’ refers to a percentage of 365 year days that have data. ‘No. of unique years with data’ is how many unique years that data is available, ‘Time period covered’ shows the percentage of data coverage (days) from the start of the record to the end (where time ranges are given in column one), and ‘Days of data available’ is the total number of unique days over the 7–8 decades that data is available. The temporal ranges in columns 3–6 occur as there are different amounts of data at different depths through the water column as per Fig. 2.

Site & Time Period	Data	Year days with data (%)	No. Unique Years with data	Time Period Covered (%)	Days of data Available
PHA Dec 1942 - Dec 2019	Bottle	99%	68–69	6–7%	1800–1863
	CTD	43–48%	25–27	1%	222–259
	Mooring	N/A	N/A	N/A	N/A
	Satellite	N/A	N/A	N/A	N/A
	Aggregated Product	99%	78	7–8%	2022–2118
	Gridded Product	100%	78	34–36%	9697–10101
NRSPHB May 1953–May 2020	Bottle	84–99%	20–53	3–6%	655–1463
	CTD	40–53%	23–26	1–2%	222–415
	Mooring (Sub-surface)	100%	12	2–8%	497–1913
	Mooring (Surface)	100%	4	3%	680
	Satellite	100%	9	6%	1532
	Aggregated Product	100%	45–63	9–19%	2164–4679
	Gridded Product	100%	56–63	25–45%	6000–10927
NRSMAI Oct 1944–Sep 2020	Bottle	87%	66	3%	742–759
	CTD	27	14–15	<1%	113
	Mooring (Sub-surface)	100%	13	10%	2806
	Mooring (Surface)	100%	3	3%	694
	Satellite	100%	9	5%	1395
	Aggregated Product	100%	77	10–13%	2959–3661
	Gridded Product	100%	77	21–28%	5941–7703
NRSROT Apr 1951–Jan 2021	Bottle	86%	40	3%	664–668
	CTD	27%	16–17	<1%	124–126
	Mooring (Sub-surface)	100%	9–12	3–12%	797–3050
	Satellite	100%	9	7%	1844
	Aggregated Product	100%	54–55	6–15%	1585–3844
	Gridded Product	100%	55–56	25–34%	6490–8543

Typically, discrete bottle samples were collected at nominal depths of 0, 10, 20, 30, 40 and 50 m depths at all sites (Table 1, Fig. 2). At NRSPHB (100 m) *in situ* samples were also taken at 75 m and 100 m (1953–1966, 1982–2010) and at 60, 70, 80, and 100 m (1966–1982) (Table 1, Fig. 2). Historically, samples were taken with a bottle attached to a marked rope, hence there is likely a margin of error in the vertical depth of the historic samples. Additionally, the surface bottle usually sampled water from about 0.5–1 m depth. Although sampling depths sometimes varied, such as at NRSPHB during the 1970s. With the expansion of the National Reference Station network in 2009 when IMOS commenced, water sampling depths and methodologies were standardised and at this time, bottle T measurements were replaced with full-depth electronic sensor profiles of T and P.

Over time these long-term NRS sites served as a catalyst for other ad hoc experiments and additional sampling occurred within the vicinity of the NRS sites. Where possible these data have also been accessed and included here, provided they pass the QA/QC criteria and are within the representative de-correlation length-scales (See scattered points in Fig. 1b–d). Hence, the aggregated data-sets provided here may also include measurements taken during one-off surveys at various depths close to the NRS sites.

It is important to note that in the case of MAI, while the site NRSMAI is nominally in 90 m of water, bottle sampling was mostly undertaken in the surface waters (0–50 m) from 1944–2008, (Table 1, Fig. 2c). Hence, at times it has been stated incorrectly that the MAI site was in ~50 m of water^6,22^, thus care should be taken. At NRSMAI after the mooring was deployed (see below) and profiling CTD casts commenced, the water column was measured full depth to ~90 m as per Table 1.

Off Perth, historically the NRSROT water sampling was in water depths of ~55 m of water^4^, but is now conducted in a water depth of ~50 m. The mooring is closer to ~60 m of water, hence, we say that the site NRSROT is nominally in ~55 m of water.

At PHA and NRSPHB electronic CTD profiles were commenced by the New South Wales (NSW) Environmental Protection Agency (EPA) in 1997 using a SeaBird Electronics SBE25 or SBE17+ (www.seabird.com). Until this time the stations PHA and PHB were in a line due east of Port Hacking NSW. In 1997, when EPA took over the sampling, two additional stations were added at PH025 and PH125 (at the 25 and 125 m isobaths respectively) and the transect was realigned to be shore normal. At this time, the PHA and PHB stations were moved 1–4 km further south and were renamed PH50 and PH100. PH025 and PH125 now also have 25 years of CTD data, which may be included in additional data products in future versions.

Profiling CTD casts were introduced at the other NRS sites sporadically at first (as far back as the 1990s) and then routinely from 2009/2010 when the sites were incorporated within IMOS. Currently electronic CTD profiles are taken using a SeaBird Electronics 19+ (SBE19+) at all NRS sites which are calibrated annually at the CSIRO calibration facility in Hobart^18^. These SBE sensors had an initial accuracy of <±0.005 °C for temperature and <0.015% of full sensor scale for pressure. Data are recorded continuously, and quality controlled using IMOS protocols, after which data are averaged in 1 m bins for the full depth of the water column. Temperature ceased to be recorded with reversing thermometer when the sites became part of the IMOS NRS network and automated profiling CTDs were introduced. It is worth noting that *in situ* bottle data is still routinely collected at all NRS sites and analysed for a range of other variables which may include salinity, total alkalinity, dissolved inorganic carbon, dissolved oxygen, and nutrient concentrations, microbes, pigments, and genetics.

### Moored temperature data (Mooring)

With the incorporation of the sites into the IMOS NRS Network in 2008/9, permanent moorings were deployed at a number of sites, including: PH100 (close to NRSPHB), NRSMAI, and NRSROT. Instrument configurations varied across the network for technical and oceanographic reasons^18^ and the main details are provided below.

A mooring has been deployed since November 2009 at Port Hacking in approximately 105–115 m of water, named PH100. The mooring is offset from the water sampling site (NRSPHB) by approximately 750 m for site security reasons. Thermistors (Aquatech AQUAloggers 520 T or 520PT) are mounted on a mooring line at 8 m intervals through the water column (nominally ranging 15–110 m), recording T or T and P every 5 minutes^19–21^. Below the sub-surface float at nominally 15–24 m was a Wetlabs water quality meter (WQM) (from May 2010 - August 2017) that consisted of a SeaBird CTD (T, S, P) and Wetlabs FLNTU measuring chlorophyll fluoresence, turbidity and dissolved oxygen concentrations^19–21^. The WQMs were set to record 60 burst samples at 1 Hz every 15 minutes. In December 2017 the WQM was replaced with an SBE37 (T, S, P) at nominally 15 m depth, and a second SBE37 was deployed 6 m above the bottom ~104 m depth. Sampling is for 3 seconds every 5 minutes. Increasingly at this site the Aquatech 520 T AQUAloggers that measure T have been replaced with Aquatech 520PT AQUAloggers that measure pressure as well as temperature at each depth. The NRS moorings all have a bottom mounted acoustic Doppler current profiler (ADCP) measuring velocity through the water column. Note that there has never been a mooring at the PHA site.

At Maria Island and Rottnest Island moorings were deployed in 2008 in approximately 90 m and 60 m of water, respectively. NRSMAI has been instrumented with Wetlabs WQMs and SeaBird SBE37s, sub-surface and near-bottom (nominally 20–25 m, and 85–90 m, respectively). NRSROT has been instrumented with a range of instruments measuring T including: Wetlabs (WQM), JFE Advantech (ACLW-USB), Seabird (SBE37, SBE39), and RBR (TDR-2050, DR-1050P, Solo). These sensors were placed on the line at a range of depths close to 22 m, 27 m, 35 m, 43 m, and 55 m, varying over time (Table 1, Fig. 2). These moorings are maintained by CSIRO.

The initial accuracy for most of the above-mentioned moored sensors is ±0.002 °C and <0.05% for T and P, respectively. For the Aquatech 520 loggers, the initial accuracy is ±0.05 °C (T) and 0.2% (P). Temperature is also measured by Teledyne RD Instruments ADCPs at each mooring site but these data are removed during the QC process due to lower quality and lack of calibration.

The depth of a temperature sensor on a mooring line can vary if there is mooring tilt due to the strength of the current. To limit uncertainty in sensor depth, typically at least 2–3 pressure sensors are fitted to each mooring per deployment (and increasingly more at PH100 where the currents are strongest). One pressure sensor is always positioned at the top of the line, often more down the line, and another towards the bottom. This gives an accurate value for the depth of the temperature record, and allows for correction of depths where pressure records do not exist. For these sensors, the IMOS ‘depthPP’ routine (https://github.com/aodn/imos-toolbox/wiki/PPRoutines#variable-transformation-transformpp) is used to estimate depth for each sensor throughout the water column.

Mooring data are mostly sub-surface, however surface floats (and sensors) have been deployed for short periods, occasionally accompanied by real time data transmission. Surface data exists at NRSMAI from 2015–2017 and NRSPHB/PH100 from 2010–2011, and 2012–2013 (Table 1, Fig. 2). At these times, T was recorded at ~0.6 m below the surface. However, inevitably the presence of a surface float makes the site both more expensive and less secure, and thus these surface expressions were removed.

After each mooring service each variable on a mooring is processed into a new netCDF file by the IMOS teams. As the deployments could range from monthly to annually, and there are sensors at a range of depths, each site could have hundreds of files (e.g. PH100 has more than 800 separate temperature data files from 2009–2021). To make this data more readily accessible IMOS have developed long time-series mooring data products (LTSP) at some sites including PH100, NRSMAI and NRSROT. These data products amalgamate and concatenate all of the individual files from each mooring deployment since the start of the IMOS period (2008/09) into one easy to access and use netCDF file per site. These single-file aggregated long time-series products were designed to improve the accessibility and usability of the mooring data and can be obtained from the AODN thredds catalog. We used these LTSP data^28–30^ as a starting point for the Mooring data incorporated into the data products presented here. As the mooring data is sampled at a range of temporal frequencies we daily-average all moored T data centred at 12:00 UTC, and incorporating data between 0:00 and 23:59 UTC. Information on the IMOS LTSPs are described at the AODN github site here: https://github.com/aodn/python-aodntools/blob/master/aodntools/timeseries_products/.

### Satellite-derived sea surface temperature data (Satellite)

Usually all the sites have some bottle or CTD sensor data near the surface, and surface mooring data is available at NRSPHB and NRSMAI for short periods as described above. However, there is insufficient *in situ* data available in the top few metres of the water column over the last decade to produce a daily surface climatology, hence, satellite-derived Sea Surface Temperature (SST) data is used to fill the near-surface gaps.

We use night-time 1 day SST data for times when mooring data is not available at the surface after January 1 2012. The data are ~2 km resolution spatially-averaged IMOS Multi-sensor night-time L3S composites of SST data^31–33^ at 0.2 m depth. Reprocessed (‘fv02’) Multi-sensor L3S files were used for the period 1st January to 31st December 2018, and near real-time (‘fv01’) Multi-sensor L3S files were used thereafter. We use night time SST composites in order to avoid possible aliasing from diurnal warming.

SST data are used within an area roughly 7 km × 9 km surrounding each *in situ* site. These distances (shown in Fig. 1) are smaller than the estimated across- and along-shelf de-correlation length scales in the East Australian Current system^34,35^. We remove the sensor specific error statistics bias^31^ from each grid cell prior to area-averaging, and add 0.17 K to convert SSTs from skin depth to drifting buoy depth (approximately 0.2 m)^31^. Only data with GHRSST (Group for High Resolution Sea Surface Temperature) ‘quality level’ flags ≥4 are used, i.e. SST data that are less likely to be contaminated by effects from cloud^31,36^. Comparisons and correlations between Mooring and Satellite data are presented in the ‘Technical Validation’ section below and Table 3.

**Table 3:** The relationships between daily nighttime area-averaged satellite-derived SST measurements and daily-averaged mooring measurements at various depth levels for the National Reference Stations at Port Hacking (NRSPHB), Maria Island (NRSMAI), and Rottnest Island (NRSROT).

Site	Mooring Depth	Comparison time period	r^2^	RMSE [°C]
NRSPHB	0.6 m	March 2010–September 2011	0.83	0.91
	15–17 m	April 2011–January 2017	0.71	1.08
NRSMAI	0.6 m	April 2015–January 2017	0.97	0.41
	18–20 m	January 2010–January 2017	0.96	0.42
NRSROT	19–21 m	November 2011–April 2014	0.90	0.47

The time periods over which satellite-derived SSTs were compared with mooring measurements are listed, along with the r^2^ (proportion of the variance explained) and root mean squared errors (RMSEs) resulting from the fits.

### Data coverage

The four sites have some of the longest ocean time-series available globally (e.g. data from 78 unique years at PHA), none-the-less there are some large data gaps (see Fig. 2). At some times and at some locations data gaps extended over 10 years (e.g. 1956–1969 at NRSROT). At NRSPHB, sampling was halted from 1960–1966 and at NRSMAI there are approximately 12 month gaps from 1966–1967, and 1988–1989. At NRSROT there is a gap from 2002–2009 where data was collected, but it is currently unobtainable. Shorter data gaps reflect the fact that bottle and CTD data were collected typically every 1 to 4 weeks, with the exact timing and frequency affected by weather and competing priorities such as for funding or the availability of staff.

When considering the total number of days available over the entire record (1940/50 s to 2021), there is between 6 and 19% data coverage across all sites and depths with a maximum of 4679 unique days of data at NRSPHB/PH100 (Table 2). Up to 12% of this total coverage is mooring data, highlighting the effect that increased temporal resolution over the past decade has on overall data coverage.

### Data products

We provide a range of data products at each site as shown in Table 4. These include an aggregation of all available QC’d time-series data, a gridded data product and daily temperature climatologies at each site. The data products are introduced briefly in this section, and the full steps taken to produce the products, and a more detailed description of the file names and format, are described below.

**Table 4:** Table showing the data products produced and their netCDF file names corresponding to the Aggregated, Gridded, and Climatology products available at the fours sites: PHA, NRSPHB, NRSMAI, and NRSROT. The data platform used (e.g. some or all of Bottle, CTD, Mooring and Satellite) and years of data coverage are shown in the file name.

Site	Data Products	File Names
PH050 /PHA	Aggregated	PH050_TEMP_1942–2019_aggregated_v1.nc
	Gridded	PH050_TEMP_1942–2019_gridded_v1.nc
	Climatology_BottleCTD	PH050_TEMP_1942–2019_BottleCTD_climatology_v1.nc
PH100 /NRSPHB	Aggregated	PH100_TEMP_1953–2020_aggregated_v1.nc
	Gridded	PH100_TEMP_1953–2020_gridded_v1.nc
	Climatology_BottleCTD	PH100_TEMP_1953–2020_BottleCTD_climatology_v1.nc
	Climatology_BottleCTDMooringSatellite	PH100_TEMP_1953–2020_BottleCTDMooringSatellite_climatology_v1.nc
	Climatology_Mooring	PH100_TEMP_2009–2020_Mooring_climatology_v1.nc
NRSMAI	Aggregated	MAI090_TEMP_1944–2020_aggregated_v1.nc
	Gridded	MAI090_TEMP_1944–2020_gridded_v1.nc
	Climatology_BottleCTD	MAI090_TEMP_1944–2020_BottleCTD_climatology_v1.nc
	Climatology_BottleCTDMooringSatellite	MAI090_TEMP_1944–2020_BottleCTDMooringSatellite_climatology_v1.nc
	Climatology_BottleCTDMooringSatellite	MAI090_TEMP_1991–2020_BottleCTDMooringSatellite_climatology_v1.nc
NRSROT	Aggregated	ROT055_TEMP_1951–2021_aggregated_v1.nc
	Gridded	ROT055_TEMP_1951–2021_gridded_v1.nc
	Climatology_BottleCTD	ROT055_TEMP_1951–2021_BottleCTD_climatology_v1.nc
	Climatology_BottleCTDMooringSatellite	ROT055_TEMP_1951–2021_BottleCTDMooringSatellite_climatology_v1.nc

#### Aggregated

We create an aggregated product at each site containing all suitable T data from the 4 platforms: Bottle, CTD, Mooring, and Satellite, where available. These data when combined represent a period from the 1940s/50 s to 2019–2021 (depending on site), and multiple depths from the surface to the bottom (Table 1, Fig. 2). All data contained in this aggregated product are either a snapshot on a particular date (e.g. bottle) or have been daily binned (e.g mooring), where time gaps between observations are indicative of data gaps in the T data sets. The aggregated products include T, depth, time, and a data source index (values of between 1 and 4 representing the different data platforms). The data source index variable is useful for selecting certain data platforms only. For example, if the user wanted to choose bottle data only, T that have a corresponding index value of 1 can be selected. These aggregated T data products are referred to as ‘Aggregated’ in Table 4 and are provided for further exploration and ease of use.

#### Gridded

We create a gridded product starting with the aggregated T data, which are then linearly interpolated in depth and time onto a regular grid (within known de-correlation length scales). The temporal resolution of this data product is daily, but gaps are present due to the interpolation technique used (see section ‘Gap Filling’ below). By gridding the data we are able to increase the temporal coverage as shown in the percentage of time covered in Table 2. The methods used are described in the ‘Data Gaps’ and ‘Optimal Depths’ sections below. These data products are referred to as ‘Gridded’ in Table 4.

#### Climatology

We define the climatological temperature as the long-term mean over multiple decades. A number of daily T climatologies are produced at each site, although it is not possible to produce all the climatologies at every site because of the differences in data sampling and availability. The simplest daily climatology produced at each of the 4 sites uses Bottle and CTD data over the full time period. The most comprehensive daily climatologies are produced using data from all 4 platforms: Bottle, CTD, Mooring, and Satellite. These climatologies are produced at the 3 NRS sites, NRSPHB/PH100, NRSMAI and NRSROT over the full time period, but is not produced at PHA/PH050 as there has never been a mooring at this site. Each daily climatology product includes the daily mean, standard deviation and percentiles. These data products are referred to as ‘Climatology_<Data Sources>’ in Table 4, where ‘Data Sources’ indicates the platform data-sets used for that particular climatology.

At NRSMAI we produce a second daily climatology that combines data from the 4 platforms for the recent time period 1991–2020 (where a 30 year climatology over a recent period is recognised as a ‘standard climatological normal’ period^37^). This was possible due to the limited number of data gaps from 1991 to 2020 and the well-mixed nature of the water column. Further, a climatology using mooring data only was produced at NRSPHB/PH100 at a range of depths using data since 2009. See Table 4 for the full list of climatology data products created at each site. The variables and depth levels used are shown in Table 5. In each case, the file name indicates what data sources were used. The steps used to create the daily climatologies are given in the following sections.

**Table 5:** The variables provided in each data product, where data source index is a number 1–4 referring to the data collection platform (i.e. Bottle, CTD, Mooring, and Satellite respectively).

Data Product	Variables	Site: [Depths] (m)
Aggregated	Temperature, Depth, Time, Data source index	PH050: [0–60]
		PH100: [0–100]
		MAI090: [0–92]
		ROT055: [0–61]
Gridded	Temperature, Depth, Time	PH050: [0–59]
		PH100: [0–100]
		MAI090: [0–99]
		ROT055: [0–60]
Climatology_BottleCTD	Mean, Standard deviation, Percentiles	PH050: [2,10,20,30,40,48]
		PH100: [2,10,20,30,40,50,60,75,99]
		MAI090: [3,10,20,30,40,49]
		ROT055: [2,10,20,30,39]
Climatology_BottleCTDMooringSatellite	Mean, Standard deviation, Percentiles	PH050: N/A
		PH100: [2,10,20,30,40,50,60,75,99]
		MAI090: [2,10,21]
		ROT055: [2,10,20,30,38]

The depth ranges over which data are provided are shown for the Aggregated and Gridded data products. For the climatologies, we show the discrete depth levels on which the climatological statistics are calculated.

### Quality control

All data-sets were quality controlled prior to being used in the data products. For IMOS surface and sub-surface mooring and CTD data collected since 2008/2009, a standardised set of quality control (QC) routines^38^ have been applied to the data using the IMOS data toolbox (https://github.com/aodn/imos-toolbox/wiki). These QC routines check for a number of data inconsistencies, such as an impossible time, depth or location, outlier values or spikes, and are described online here: https://github.com/aodn/imos-toolbox/wiki/QCProcedures. QC flags are created following the UNESCO Intergovernmental Oceanographic Commission (IOC) protocol flags^18,38^. We use data with corresponding QC flags ‘Good data’ and ‘Probably good data’ here.

Historically the bottle data were initially quality controlled by CSIRO. We suspect that quality control best practices have changed over time at the NRS sites, related to continuing advancement of ocean sciences. Currently CSIRO use up to 255 QC flags, with each one specifying whether the data is ‘Good’, ‘Suspect’, or ‘Bad’ quality or whether ‘No QC’ has been applied, accompanied with a specific data issue (e.g. ‘Hardware error’, ‘anomalous spike’ etc.). More information is available at https://www.marine.csiro.au/data/trawler/download.cfm?file_id=76. All bottle data that we obtained were originally flagged as ‘Good’, however additional QC was performed to identify the following issues^17^:

- data outliers outside of a 4 standard deviation threshold when considering the entire T data source record at a site
- data at a location that is considered too shallow or too deep to be representative of the sites used here
- profiles that are outside a defined spatial proximity to the nominal reference station location based on known de-correlation length scales on the southeastern Australian shelf^34^, and
- profiles with more than 30% of data flagged previously.
  The IMOS-developed Multi-sensor night-time L3S SST data include quality level (QL) flags following GHRSST conventions^36^, but “remapped” to account for the different methods used to derive QL of the various SST data streams ingested into the Multi-sensor L3S product. See Appendix A in Griffin *et al*. (2017) for further details on how the remapped QL is derived. The QL flags range between 0 and 5, with QL flags equal to 0 and 1 indicating missing and invalid data (e.g., too close to land), respectively. QL flags 2 to 5 represent the likelihood of the SST value to be affected by cloud, with a QL flag equal to 5 representing the highest quality (least cloudy) data and QL flag equal to 2 the worst quality (most likely cloudy) data. In addition, L2P flags were utilised enabling the selection of SST data based on various quality indicators (e.g. atmospheric quality, swath characteristics, land, ice, etc.). We used only SST data that corresponded with QL = 4 and 5 values, and excluded data measured using microwave, and/or associated with potential atmospheric aerosol contamination.
  In addition to the listed QC checks, the aggregated data-sets were analysed manually for any inconsistencies, incorrectly flagged data, or data considered as ‘suspect’. Some examples of ‘suspect’ data include:
- profiles where the deepest T measurement is deeper than the known bathymetry at that location
- remaining outliers that were not detected when considering the entire record, but present when selecting different data subsets (e.g. month-by-month comparisons and depth comparisons)
- satellite SST that are cooler than the nearest sub-surface mooring data by more than 0.05 °C
- mooring T at one nominal depth being warmer than T at a shallower nominal depth
- T measured by current meters (ADCPs), which often have a clear offset, when compared with data from other nearby sensors deployed at the same time.

The climatology statistics at NRSROT were warm biased by observations during the strong 2011 La Niña event^39^. Hence, data were excluded between 1 January and 1 June 2011 for calculating the climatology, but are present in the other NRSROT data products (see section ‘Data Products’ above).

### Gap filling

Noting that the aggregated data-sets have gaps ranging days to years, to potentially reduce the effect of missing data on the derived climatologies, T is linearly interpolated in time and depth prior to calculating the climatological statistics. To avoid using interpolated T too far away from an observation in space and time (e.g. in between monthly sampling), and thus considered likely erroneous, any interpolated data greater than defined spatial and temporal limits were not used. These limits are based on de-correlation length scales that were determined using auto-correlation analysis[^17^, their Fig. 4] and are listed in Table 6 for each site.

**Table 6:** The de-correlation length scales for depth and time estimated using auto-correlation analysis at the three NRS sites: NRSPHB, NRSMAI, and NRSROT.

Site	Depth Threshold	Time Threshold
NRSPHB	8 m	2.5 days
NRSMAI	6 m	2.5 days
NRSROT	3 m	3 days

Data collected since 2008/2009 has a higher temporal resolution than data collected prior, meaning that temporal interpolation over the last decade was not as necessary as over the preceding decades. On days when both observations and temporally interpolated data exist, the interpolated data are not used. Linearly interpolating the aggregated data increased the temporal coverage to between 21% and 45% when considering all sites and depths (Table 2). As there are still some gaps in the data-sets, climatology data product users should keep in mind the potential uncertainty of climatology statistics.

### Optimal depths

The depth bins (1 ± 2 m) through the water column where we calculate the climatological statistics are chosen using the vertical data distributions at each site^17^. For each climatology produced, the availability of data from two to three data sampling platforms is compared over the full depth of the water column to determine the depths where data availability is at a maximum, which we refer to as the optimal depths. In most cases, depth bins that contain data from at least two data platforms are used and preference is given to depth bins that have bottle data over other data platforms. This does not apply to the mooring only product - see ‘Data Products’ section above. Potential optimal depths are evaluated for the percentage of the year covered by, and the number of unique data years available in each depth bin before proceeding to calculate the climatologies (See Table 2).

After a climatology is produced at these optimal depths following the steps described below, the climatological statistics are then interpolated over depth at standard depth targets. For example, at NRSPHB climatology statistics are available at 2 m, then every 10 m until 60 m, 75 m and then finally at 99 m, with the first and last depth targets being the same as the optimal depths. Hence, the climatology statistics were produced at depths where data availability is high, but are provided at convenient regular depth targets (Table 5).

### Creating the climatologies

**Fig. 3:**
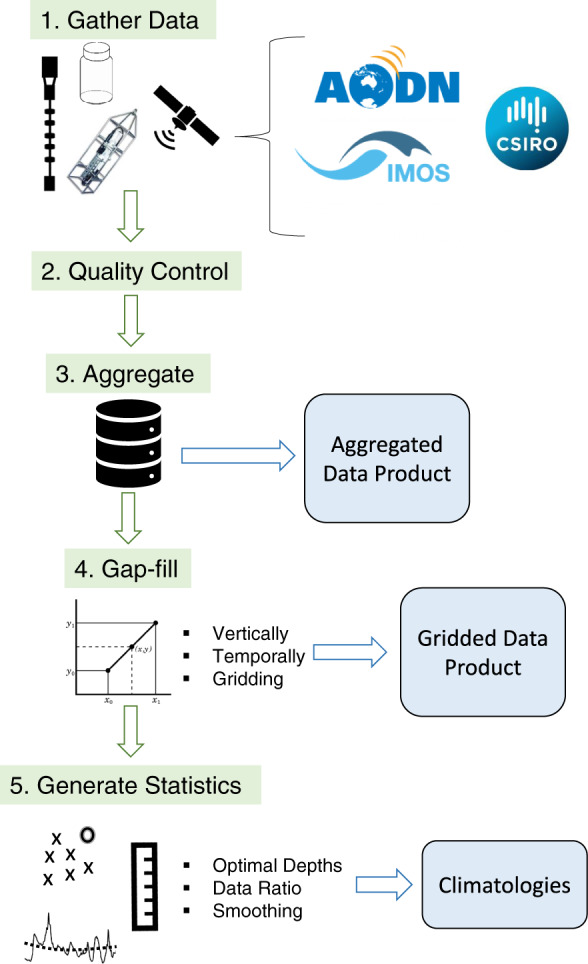
A schematic diagram showing the steps undertaken to create the aggregated, gridded and climatology data products.

To create the climatologies we use a combination of steps^7,17^ that are listed briefly here and illustrated in Fig. 3. Steps 1–3 refer to gathering the many different data-sets from the 4 platforms, conducting data QC, and aggregating the data into the ‘Aggregated’ data product. This is followed by gap filling and interpolation as described above to generate the ‘Gridded’ data product. In step 5 we first find the optimal depths for the climatology statistics using data availability throughout the water column. Following this, for each of the optimal depth bins and day of the year we incorporate daily T within a time-centered moving window of 11 days, taking into consideration dates when more than one data source is available. We account for differences in sampling frequency and temporal range with the various data platforms by using a ratio of data from the different sources^17^. For example the climatology derived from data from all 4 data platforms (Bottle, CTD, Mooring and Satellite) uses a bottle to mooring ratio of 6:1 at NRSMAI and NRSPHB, and 5:1 at NRSROT (see Technical Validation below for more information). In this way we avoid sampling biases due to changes in spatial and temporal resolution from one decade to another^17^ (i.e. the climatology would be biased towards the most recent warm decade if we used all the mooring data from the past decade). Finally, we derive the climatology statistics at optimal depths, smoothed using a moving average window of 31 days^7^.

**Fig. 4:**
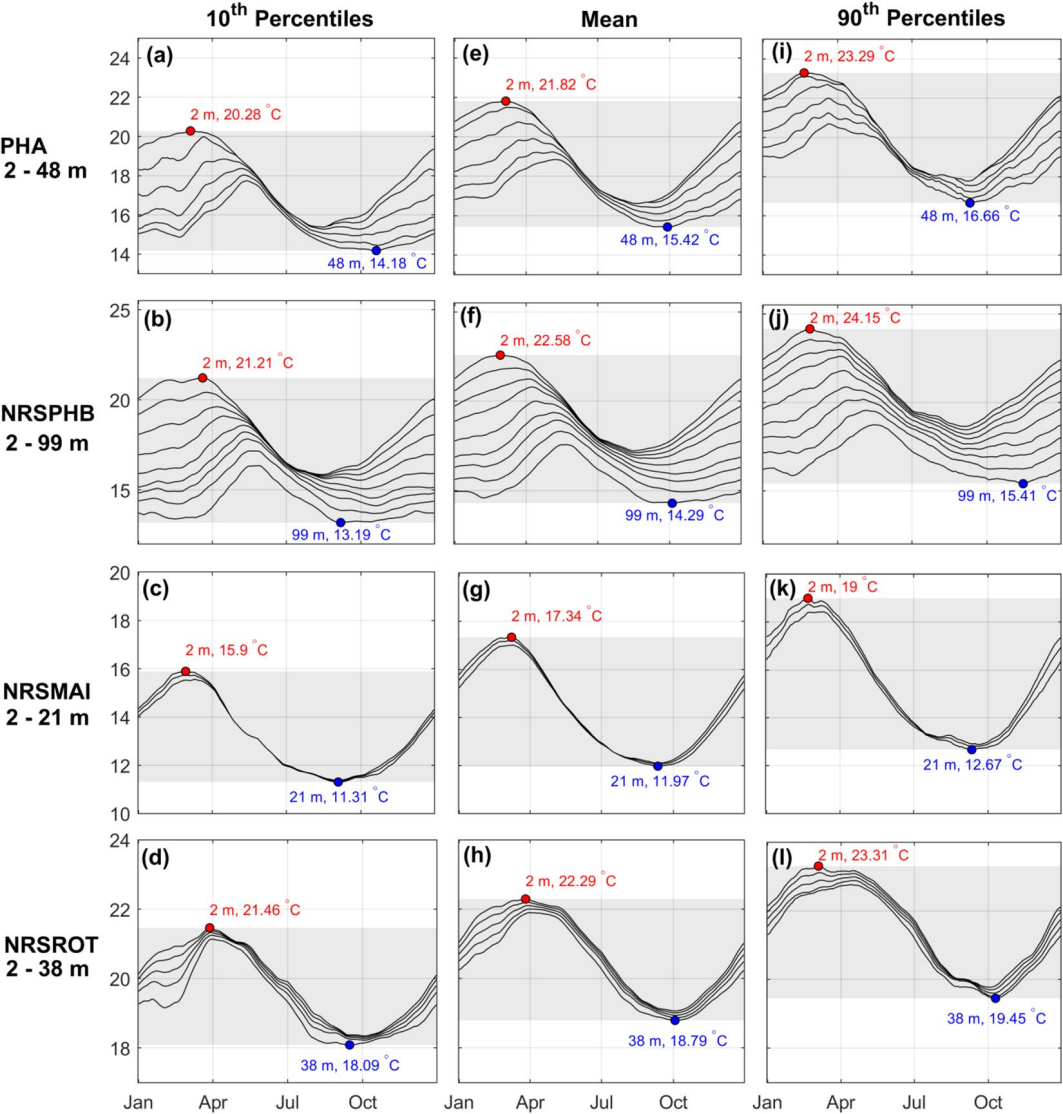
Plots showing the temperature statistics from each climatology for the four sites: PHA, NRSPHB, NRSMAI, NRSROT showing the 10^th^ percentiles (**a**)–(**d**), means (**e**)–(**h**), and 90^th^ percentiles (**i**)–(**l**). The statistics shown here were calculated using all available data sources from the 4 platforms (Bottle, CTD, Mooring and Satellite) over the full time period (1940/50 s to 2020), except for PHA which includes Bottle and CTD data only. The grey shading in each panel shows the temperature range (noting various y-axis limits), with maximum (red point/text) and minimum (blue point/text) temperatures highlighted for the shallowest and deepest depths, respectively. Depths where the statistics are calculated are shown in Table 5.

The multi-platform climatologies at each site are shown in Fig. 4. The middle column shows the mean temperature by day of the year at each site and chosen depth (where depth is shown in Table 5). Also shown are the 10^th^ and 90^th^ percentile temperatures.

## Data Records

### Accessing the data products

The data products are archived at the Australian Ocean Data Network and can be accessed under the Creative Commons 4.0 licence here: http://thredds.aodn.org.au/thredds/catalog/UNSW/NRS_climatology/Temperature_DataProducts/catalog.html

Any and all use of the data products provided here must include:

- a citation to this paper,
- a reference to the data citation as written in the netCDF file attributes and as follows: Roughan, M. *et al*. (2022)^40^ “Multi-decadal ocean temperature time-series and climatologies from Australia’s long-term National Reference Stations”, Australian Ocean Data Network, 10.26198/5cd1167734d90.”
- the following acknowledgement statement:

*Data was sourced from Australia’s Integrated Marine Observing System (IMOS) - IMOS is enabled by the National Collaborative Research Infrastructure Strategy (NCRIS)*.

Any updated data products, and/or potential new products (e.g. at other sites or using other ocean variables) will be hosted at the same location. Therefore it is advised that data users seek the latest data product version.

### File naming convention

We use site names that include the approximate depth of the site, hence, NRSPHB/PH100 is shortened to ‘PH100’, and ‘MAI090’ and ‘ROT055’ are used instead of NRSMAI and NRSROT, respectively. The files are saved in netCDF format with the following naming conventions:

For the Aggregated and Gridded products:<site name>_<variable>_<year range>_<data product type>_<version>.nc

For the Climatologies:<site name>_<variable>_<year range>_<data platforms used>_climatology_<version>.nc

For example at NRSPHB/PH100 the filename for the aggregated time-series is ‘PH100_TEMP_1953–2020_aggregated_v1.nc’ and the filename for the climatology using data from the 4 data platforms between 1953 and 2020 is ‘PH100_TEMP_1953–2020_BottleCTDMooringSatellite_climatology_v1.nc’.

### Data formats

The netCDF files have been created to comply with the Climate Forecasting conventions and metadata standards (CF, Version 1.6, https://cfconventions.org/) and IMOS netCDF conventions (version 1.4, http://content.aodn.org.au/Documents/IMOS/Conventions/IMOS_NetCDF_Conventions.pdf).

The variables contained in each file and their filenames are summarised in Tables 4 and 5, and their metadata are summarised in Table 1. Each variable contained in the aggregated file is an array of equal length containing the concatenated data-sets. These arrays have the dimension ‘OBSERVATION’ which is monotonically increasing, and is similar to the IMOS mooring LTSP files. Variables, such as ‘TIME’ or ‘DEPTH_AGG’, might seem disordered at first glance because of the way the individual profiles have been concatenated together. However, each variable array is linked by the ‘OBSERVATION’ dimension and are easily plotted or sliced into subsets. The gridded and climatology products contain variables presented as 2D matrices with rows and columns representing depth and time, respectively. This enables easy selection of data by depth or time. For example if using the gridded product, selecting all columns (TIME) and the first row (DEPTH) would provide all available T data over time at the surface.

## Technical Validation

When developing the methodology for combining multi-platform T data into long time-series for a robust climatology we went through a rigorous validation of the methodology. Using data from NRSPHB/ PH100 the steps used for combining the different data types were established, tested and published^17^. The goal was to ensure that when the data from different sampling frequencies over different decades was combined, we limited the potential biasing of climatology statistics, e.g due to the increased number of samples during recent (warmer) decades. In addition, the methodology paper has been accepted into the Oceans Best Practices repository having undergone further peer review (https://repository.oceanbestpractices.org/handle/11329/1393).

### Comparison with synthetic temperature data

To test the approach synthetic data were generated for the NRSPHB/PH100 site with similar statistical properties to the observed T data between 1953 and 2019^17^. Inter-annual and inter-decadal variability was added to the synthetic data. The synthetic data were then used to test the methodology accounting for the sampling differences between bottle, CTD and mooring data in time and space. The effects of changes in temporal sampling resolution (e.g. how best to combine 5 minute moored data collected in the last (warmer) decade with approximately monthly bottle data from 6 decades), the ratios of different data sources, and the use of a time-centred moving window to create the climatology were investigated and published^17^.

The final methodology was chosen based on the RMS errors calculated between the synthetic reference statistics and the statistics generated for the different data combinations^17^. It was through this rigorous approach that we chose daily mooring data, a time centered moving window, and a bottle to mooring year ratio of 6:1 (at PH100). Increasing the time centered window by more than ±2 days had little effect on the resulting RMSEs between the synthetic reference statistics and the statistics generated for different data combinations. Here, we choose a window of ±5.5 days for the climatology to be consistent with the methodology^7^ commonly used in marine heatwave studies^8,15,41^.

### Comparison with satellite-derived temperature data at the surface

To augment the time-series at the surface for sites and times when we have no *in situ* surface data we included multi-sensor night time satellite-derived SST from January 2012 onward (as described above). Table 3 shows validation statistics (R^2^ and RMSE) between satellite-derived and *in situ* T (mooring). Surface satellite data agreed relatively well with moored T data; sub-surface, r^2^ = 0.71 to 0.96 and surface r^2^ = 0.83 to 0.97. The lowest r^2^ values, and highest RMSEs between satellite and mooring data were estimated at NRSPHB. Of the 3 NRS sites where we used satellite and mooring data, this site is the most stratified (Table 1), and the most dynamic, being affected by coastal upwelling/downwelling^42,43^, river outflow^1^, and the East Australian Current eddy field^44,45^.

### Diurnal variability

The effect of diurnal temperature cycles on the calculated climatology statistics was explored when developing the methodology^17^ that we use here. Using mooring data at NRSPHB, diurnal cycles were shown to be generally less than 0.4 °C, but between 0.4 and 0.7 °C at some depths^17^. The most distinct diurnal cycles were observed in Summer and Autumn. Further the potential bias on the mean and 90^th^ percentiles was estimated when using mooring data collected in the morning and those measured throughout the day and found to be between 0 ± 0.4 °C and 0.3 ± 0.5 °C^17^.

### Sampling-related statistical overlap

A temperature inversion could occur in a particular dataset (e.g. through slow responding electronics), that would likely be flagged as ‘bad’ in the QC process. But it could also occur in the climatological statistics through the merging of multiple data sets from different platforms over multiple decades. In this case, data availability over depth and time varied and therefore, in some instances caused overlap between the climatological statistics vertically through the water column. This means that on occasion, climatological T statistics at one depth could have been slightly warmer than (overlapped) those at the depth level above. This was seen to occur on a handful of occasions at sites where the water column was well mixed in late Autumn and Winter. For days when this occurred, if the T difference between statistics at two neighbouring depths was between 0 and 0.1 °C, the intruding statistics at the deeper climatology depth were replaced by the statistics calculated at the neighbouring depth above. If the T difference was higher than the 0.1 °C threshold, then the statistics at the deeper climatology depth were flagged and excluded from the climatology data product. Statistical overlaps typically occurred over short periods of time, and only for certain depths/statistics per climatology data product.

## Usage Notes

The data products described here from the 4 long-term ocean data sites around Australia can be used to investigate T regionally, temporally, and through the water column. For example, the data products can be used to identify mean T, trends, and anomalies and extremes including marine heatwaves and cold spells. As the data products include T at a range of depths through the water column, the vertical T structure, its trends and corresponding variability can be investigated at a range of depths through the water column on any given day of the year.

We provide basic scripts in Matlab, Python and R demonstrating how to download and load the data products, produce plots, and export the data as CSV files. These scripts are available online at figshare^46^ and are made available under a Creative Commons Attribution 4.0 International license (CC BY 4.0).

## Data Availability

The code for the IMOS mooring toolbox used to quality control IMOS data can be accessed here: https://github.com/aodn/imos-toolbox. The code used to aggregate the mooring time-series data is available here: https://github.com/aodn/python-aodntools/tree/master/aodntools/timeseries_products. MATLAB, Python, and R tutorials have been created to help users download, load, plot, and export data (as CSV files) contained in the products. These are publicly available under a Creative Commons Attribution 4.0 International license (CC BY 4.0) on line at figshare^46^.
